# Coping with intrasexual behavioral differences: Capture–recapture abundance estimation of male cheetah

**DOI:** 10.1002/ece3.4410

**Published:** 2018-07-30

**Authors:** Sarah Edwards, Manuela Fischer, Bettina Wachter, Joerg Melzheimer

**Affiliations:** ^1^ Evolutionary Ecology Leibniz Institute for Zoo and Wildlife Research Berlin Germany; ^2^ Centre for Wildlife Management University of Pretoria Pretoria South Africa

**Keywords:** abundance, capture–recapture model, cheetah, heterogeneity, intrasexual behavioral differences

## Abstract

Population estimates are a fundamental requirement of ecology and conservation. While capture–recapture models are an established method for producing such estimates, their assumption of homogeneous capture probabilities is problematic given that heterogeneity in individual capture probability is inherent to most species. Such variation must be accounted for by abundance models; otherwise, biased estimates are risked.
Here, we investigate the performance of four types of heterogeneity models for estimating abundance of male cheetah *Acinonyx jubatus*, a species with two distinct spatial tactics of territorial and nonterritorial (floater) males. The differences in spatial movements of territory holders and floaters are expected to result in intrasexual heterogeneous capture probabilities. Four heterogeneity models were used to model male abundance at five territories in central Namibia; (a) a spatial tactic model, (b) a finite mixture model, both run in program MARK, (c) a floater‐only model, and (d) a heterogeneity M_h_ model, both run in the program CAPTURE. Camera trap data of cheetah, taken at frequently visited marking trees, were used to derive true abundance. Model results were compared to the true abundance to assess the accuracy of estimates.
Only models (a), (b), and (c) were able to consistently produce accurate results. Mixture models do not require prior knowledge regarding spatial tactic of males, which might not always be available. Therefore, we recommend such models as the preferred model type for cheetahs.
Results highlight the potential for mixture models in overcoming the challenges of capture probability heterogeneity and in particular their use with species where intrasexual behavioral differences exist.

## 1 INTRODUCTION

Estimates of population size are an important and fundamental requirement of ecology and the conservation management of wildlife (Baker, 2004; Otis, Burnham, White, & Anderson, 1978), requiring robust, reliable, and efficient methodology (Harmsen, Foster, & Doncaster, 2010). Unbiased and precise estimates are especially essential for species under threat, as well as for exploited species, for which overestimates of abundance could lead to unsustainable takeoff levels (Baker, 2004). Factors such as large geographical ranges and low detection probability often mean entire populations cannot be surveyed simultaneously. Thus, surveys usually seek to monitor a proportion of the population, which requires methods accounting for imperfect detection of individuals (Sollmann et al., 2013; Williams, Nichols, & Conroy, 2002). Capture–recapture models are used in many ecological studies (Foster & Harmsen, 2012), including photographic capture–recapture sampling methods, which were originally developed to estimate tiger *Panthera tigris* density (Karanth & Nichols, 1998). They are one method to estimate animal abundances if species can be individually identified. These models are frequently used in combination with camera traps or other noninvasive devices such as hair snares, to repeatedly sample marked individuals at fixed locations (Otis et al., 1978; Royle, Nichols, Karanth, & Gopalaswamy, 2009). Individual encounter histories are then used to calculate capture probability, such that the abundance estimate is regarded as the size parameter of a binomial distribution (Royle, Chandler, Sollmann, & Gardner, 2014).

One of the major challenges facing estimation of population size is the heterogeneity in capture probability among individuals (Boulanger, Stenhouse, & Munro, 2004), because equal capture probability is a general assumption of traditional capture–recapture models (Krebs, 1999). Violation of the assumption usually leads to biased abundance estimates (Burnham & Overton, 1978; Cubaynes et al., 2010). Significant variation in capture probability has been suggested as the reason for negatively biased abundance estimates in Hawaiian monk seal *Monachus schauinslandi* (Baker, 2004) and painted turtles *Chrysemys picta* (Koper & Brooks, 1998), in comparison with true abundance.

Heterogeneity in capture probability has been suggested to be inherent in any animal population (Lebreton, Burnham, Clobert, & Anderson, 1992) and may arise for a multitude of reasons (Harmsen et al., 2010), for example, differences in sex, for example, jaguar *Panthera onca* (Sollmann et al., 2011), age, breeding status, for example, southern right whale *Eubalaena australis* (Carroll, Steel, & Baker, 2013), behavior, and social status of individuals, for example, coyote *Canis latrans* (Larrucea, Brussard, Jaeger, & Barrett, 2007). In addition, heterogeneity may arise when the home range of the species is large in comparison with the surveyed area. Such a factor may result in the study area containing only a partial home range of some individuals, with these individuals experiencing exposure to less camera traps than others (Oliver, Morgan, Durant, & Pettorelli, 2011; Royle et al., 2009).

Intrasexual heterogeneity in capture probability is expected for species in which differences in social status or behavior exist within the sexes, which may result in different use of a study area (Perret, Pradel, Miaud, Grolet, & Joly, 2003). Although capture–recapture methods have been developed for populations in which transience or temporary emigration occurs, these models were primarily designed for survival estimation, rather than abundance estimates (Pradel, Hines, Lebreton, & Nichols, 1997). Otherwise, when abundance is estimated, models only produce resident abundance estimates or permit raw data entries for transients with only one capture during the survey period (Conn, Gorgone, Jugovich, Byrd, & Hansen, 2011). However, for species in which transient or nonterritorial individuals are expected to be captured more than once, these models are not appropriate.

Cheetah *Acinonyx jubatus* is one species which exhibits intrasexual behavioral differences of adult males after they dispersed from their natal home range and established in a new area (Caro, 1994; Melzheimer et al., 2018). Adult males are either territory holders occupying small territories (in Namibia: 379 ± 161 km^2^ [mean ± standard deviation]) or floaters ranging over large areas (in Namibia: 1,595 ± 1,131 km^2^, Melzheimer et al., 2018). Territorial males mark and defend their small territory, while floaters roam over much larger areas which they do not actively defend (Melzheimer et al., 2018). Territorial male cheetahs mark at prominent landmarks (Caro, 1994), which in southern Africa are typically trees with low, sloping branches (Marker‐Kraus, Kraus, Barnett, & Hurlbut, 1996). In Namibia, territorial males marked such trees in approximately 94% of their visits to these trees with urine or feces. In contrast, floaters were never or rarely recorded scent marking at such trees and, rather, visit trees to sniff markings of territorial males (Melzheimer et al., 2018; Wachter et al., 2018). Territory ownership is usually the final stage in the life history of a male cheetah; however, not all individuals will become territorial, some will remain floaters throughout their lives (Melzheimer et al., 2018). Such differences in the spatial ecology of adult males are likely to result in differential use of a survey area, thus creating heterogeneity in capture probability.

Cheetah has been identified as a species in need of accurate and precise population estimates due to its rapid decline (Broekhuis & Gopalaswamy, 2016). The species is currently occupying only 9% of its historical range, and a total global population of approximately 7,100 individuals is estimated with the majority of the animals occurring in southern African (Durant et al., 2017; Weise et al., 2017).

Here, we aim to identify the most reliable model for producing male cheetah abundance estimates within single territories, by comparing the results of a number of models accounting for heterogeneity against known abundance estimates from five territories in central Namibia. Population estimates across larger landscapes can then be calculated on the basis of such smaller units, that is, the territories. Producing accurate abundance estimates at a territory level is therefore crucial for subsequent analyses and provides a first step in the process of producing accurate population estimates.

## 2 METHODS

### 2.1 Study area

**Figure 1:**
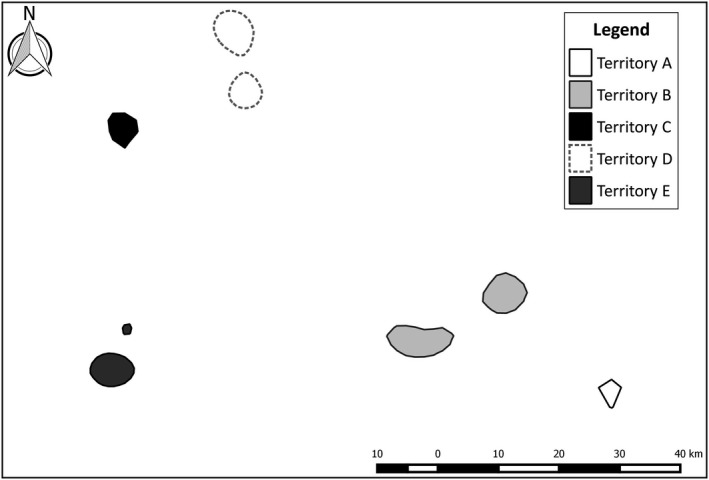
Five cheetah territories, represented by 50% kernel density polygons derived from territorial male spatial data, used for estimating male cheetah abundance

Data for this study were collected from September 2011 to March 2012 at five male cheetah territories, A to E, located within the east‐central highlands of Namibia, approximately 150 km east of the capital Windhoek (Figure 1). All territories were located on commercial game and cattle farms, in habitats dominated by shrub savannah (Barnard, 1998), with an average annual rainfall of 370 mm (http://en.climate-data.org/location/904176/). The five territories were chosen as those best known from a long‐term study of cheetahs in the area, which included the use of camera traps to detect marking behavior for a previous study (Melzheimer et al., 2018; Wachter et al., 2018). Due to the long‐term monitoring of these specific territories, the identities of all territorial and floater males were known.

### 2.2 Camera trap methods

**Figure 2:**
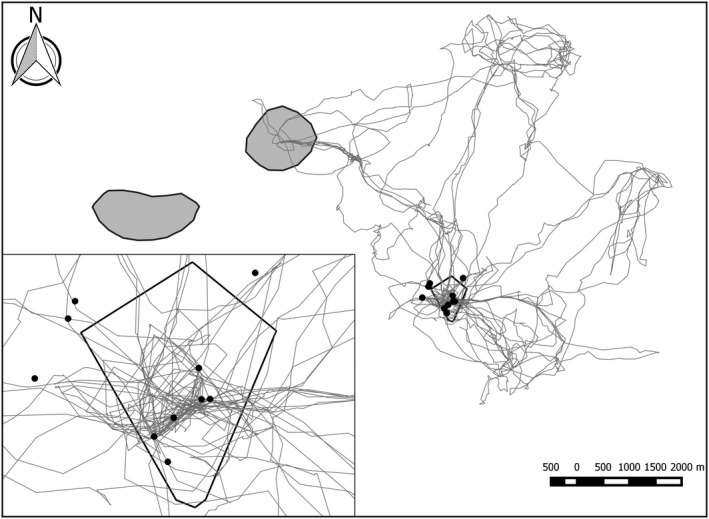
Spatial data movement from a single floater male during the survey period (September 2011–March 2012). Insert showing the movement of the same floater male with reference to the 10 camera traps in territory A

To maximize capture probability, camera trap stations were placed at marking trees, which represent predictable locations of cheetah activity (Caro, 1994). Marking trees were identified using the spatial data of territorial males which were shown as clusters of locations when plotted. Male cheetahs were captured in box traps at marking trees and immobilized as described in Thalwitzer et al. (2010). Single males were always collared with a GPS collar (VECTRONIC Aerospace GmbH, Berlin, Germany; e‐obs GmbH, Grünwald, Germany), and when coalitions of males were captured, at least one male was fitted with a GPS collar and the other(s) with a VHF collar (Advanced Telemetry Systems, Isanti, Minnesota, USA). Due to the higher frequency of locations obtained from GPS collar (up to one position every 15 min) than VHF collars, only GPS positions were used to identify the clusters representing marking trees. Identified marking trees were visited in the field to assess the number and freshness of scats. We assumed that the number of scats was positively related to the frequency of cheetah visits and scat freshness identified recent cheetah activity. Hence, the marking trees with a combination of both fresh and numerous scat were chosen for camera trap placement. The final ten marking trees used for camera trap placement were spread across the home range of the territorial animal, defined as the 95% kernel density polygon derived from the spatial data of the territorial animal occupying that territory. Most marking trees fell within the core of the home range; the 50% kernel density polygon (Figure 2; territory A shown as an example). For each territory, a 28‐day survey length was used, which falls within the recommended closed period for large felidae (Karanth & Nichols, 1998). Some territories were survey simultaneously, others consecutively, which was due to the number of camera traps available. This resulted in a total survey period of 95 days. The program CloseTest (Stanley & Burnham, 1999) was used to test for demographic closure. Each camera trap station consisted of two Reconyx PC900 HyperFire camera traps (Reconyx Inc, Holeman, Wisconsin, USA), positioned opposite each other, with enough offset to eliminate flash interference. Camera traps were positioned within 3–5 m from a marking tree facing the tree and mounted on poles approximately 70–90 cm above ground. Traps were programmed to be active 24 hr a day, taking three photographs per trigger, with no delay between triggers. Camera stations were revisited every 7–10 days, to change SD cards and batteries and check for camera functioning.

### 2.3 Comparison of model type and performance

Detection histories for all adult male cheetah at each territory were constructed, using their unique pelage pattern for individual identification (Caro, 1994), and the presence of testes for identification of sex. Male cheetahs roam solitarily or in coalitions of two or three, rarely four males (Caro, 1994). Coalition members were treated as one unit, resulting in one detection history per unit, as male coalitions are stable, and the close proximity of coalition members results in identical movement patterns (Caro, 1994). Detection histories consisted of seven sampling occasions each of 4 days in length (Supporting Information Figure S1). A 4‐day sampling occasion was chosen because existing movement data of collared cheetah individuals indicated that floater males were present in a territory every 7–10 days (Fischer, 2012). Therefore, seven 4‐day sampling occasions should ensure each floater male is recaptured at least once during the survey period of 28 days. A closed population, that is, a population which remains constant in size and composition throughout the period of investigation is a crucial assumption for the model types compared here (White et al., 1982). It was considered that the assumption of a demographically closed population was met as a trapping period of 28 sampling days was short enough that mortality, birth, and migration in and out of the population was not expected. The movements of the floater males in and out of a sampled territory could be interpreted as the population not being geographically closed. However, floaters do have stable home ranges and include the small territories as part of their large home range (Figure 2). Therefore, we consider the population as closed.

Male cheetah abundance at each territory was estimated using four heterogeneity model types: (a) a Huggins type covariate model, that is, a spatial tactic model, run in program MARK; (b) a Pledger model (Pledger, 2000), that is, a finite mixture model, henceforth referred to as mixture model, ran in program MARK; (c) a “floater‐only” model, in which only floater males were included in the detection histories run in program CAPTURE; and (d) a heterogeneity *M*_h_ model with the jackknife and Chao estimators, run in program CAPTURE. The spatial tactic model was run with spatial tactic coded as an attribute group affecting both capture and recapture probabilities, rather than a traditional Huggins model, because the former calculates abundance in the likelihood (Cooch & White, 1999), thus allowing direct comparisons with mixture models to be made (Williams et al., 2002). Mixture models were run using two mixtures of capture and recapture probabilities; one for territory holders and one for floaters (White, 2008). Mixture models do not require the spatial tactic of each male to be identified. In addition to an abundance estimate, these models produce an estimate of *π*, the probability of any individual in the population being, in this case, a floater. Four predefined models were ran for the spatial tactic and mixture models: (a) *M*_o_ (null model in which all capture and recapture probabilities are equal); (b) *M*_h_ (heterogeneity model with two mixtures, each of equal capture and recapture probabilities for territory holders and floaters, respectively); (c) *M*_b_ (behavioral model with one mix of different capture and recapture probabilities, but territory holders and floaters having the same capture and recapture probability); and (d) *M*_bh_ (behavior and heterogeneity model with two mixtures of capture and recapture probabilities, plus a behavioral response, which considers a differential response if the individual has been previously captured, that is, trap‐happy or trap‐shy (Anile, Amico, & Ragni, 2012). In addition, for spatial tactic models, combinations of *M*_o_ and *M*_b_ for territorial and floater males were run. Model fit was ranked using Akaike information criterion (AIC) values (Akaike, 1973), adjusted for small sample size (AICc) to indicate the level of support given to each model (Burnham & Anderson, 2002).

The program CAPTURE, accessed via MARK, was used to estimate male cheetah abundance using the floater‐only model and the heterogeneity *M*_h_ model with the jackknife and Chao estimators for both models, respectively, with Chao models being theoretically more robust to small sample sizes (Boulanger et al., 2004). For these two models, the *M*_o_, *M*_b_, and *M*_h_ predefined models were run. When running floater‐only models, CAPTURE's model selection test was used to select the most appropriate model from the candidate set of *M*_o_, *M*_b_, and *M*_h_ (both jackknife and Chao) by ranking model fit (Burnham & Anderson, 2002).

Spatial tactic models require that each cheetah unit is identified as having either a territorial or floater spatial status. Spatial tactic was determined by examination of spatial data, with spatial tactic coded as a dummy variable. During the survey period, the identity of the territorial individuals at territory D was uncertain, because two different male coalitions were scent marking; therefore, for this territory only, the mixture model and the CAPTURE's heterogeneity *M*_h_ model were used, given that these models do not require identification of spatial tactic.

### 2.4 True male cheetah abundance at each territory

GPS data from all collared floaters (*n* = 8) within the study area were used to validate that each floater entering a territory core of territory males was captured on camera trap, and thus, true abundance was known for each individual territory. GPS data of collared floaters that entered a territory core were compared with sampling events to check that all floaters were captured on camera trap each day and every respective sampling occasion they were present within a territory. As this was verified (see Section 2.1), we assumed that also all VHF‐collared and noncollared floater males were captured each time they entered a territory. Some marking trees were located outside of the 50% kernel density polygon (Figure 2), due to the fact that monitored marking trees were selected based on the number and freshness of cheetah scats present. However, this did not influence analysis, as peripheral trees were also included in comparison of sampling events and GPS data (Figure 2).

## 3 RESULTS

### 3.1 Camera trap statistics

Cheetah photographs were classified into independent events, using a criterion of a minimum of 30 min between consecutive photographs of the same individual (O'Brien, Kinnaird, & Wibisono, 2003), giving a total of 603 cheetah events for the study. Females accounted for 24 (3.98%) events, and 27 (4.48%) events were unidentifiable to the individual level. Thus, these events were excluded from analysis and the remaining 552 events used. A total of 36 floater males were recorded, four of which were recorded at two territories (Figure 2; one floater shown as an example), and one was recorded at three territories. Cheetah was detected at eight to ten marking trees per territory. Camera trap success within a territory ranged from 13.21 to 35.71 events/100 trap nights for territory holders, from 7.86 to 20.07 events/100 trap nights for floater males within territories, and from 24.63 to 56.82 for all males combined within territories. Capture probability using a 4‐day sampling occasion ranged from 0.85 to 1.00 for territory holders males and from 0.29 to 0.36 for floater males. Performance of the CloseTest supported the assumption of population closure for all territories, with the exception of territory A (*χ* ^2^ = 12.59, *df* = 5, *p *=* *0.03).

### 3.2 True male cheetah abundance at each territory

During the study, spatial GPS data showed that all collared floaters were present within territory cores on a total of 95 days. Camera traps detected individuals within the cores on 91 of the 95 days, when using a temporal resolution of 24 hr, giving a detection probability of 95.79%. When using a 4‐day sampling occasion, as used in the capture–recapture models, every time a collared floater entered a territory it was captured on camera trap during the respective sampling occasion, resulting in a 100% detection probability. The 100% detection probability, for detecting floaters entering a territory core, therefore justifies the critical assumption that the true abundance of individuals visiting a territory is known and thus allows meaningful comparisons of true abundance and capture–recapture model estimated abundance to be made in order to assess their performance.

### 3.3 Comparison of model type and performance

The spatial tactic model *M*_o_ (Territorial), *M*_b_ (Floater) was the best fitting model for each territory (Table 1). This model suggests equal capture and recapture probabilities for territorial males and different capture and recapture probabilities for floater males. The top fitting mixture model varied between territories, with the behavior and heterogeneity model (*M*_bh_) being the best fit for two territories (A and B), while the heterogeneity model (*M*_h_) was the best fitting model for two other territories (C and E, Table 1). When using program CAPTURE to select the most appropriate model for the floater‐only approach, the null model (*M*_o_), was always ranked as the best fitting. For a full comparison of all predefined spatial tactic and mixture model abundance estimates, see Supporting Information Table S1.

**Table 1:** Comparison of best fitting spatial tactic and mixture models for each territory

Territory	Top spatial tactic model	AIC_c_ spatial status	Parameters	Top mixture model	AIC_c_ mixture	Parameters	Delta AIC_c_
A	*M*_o_ (Territorial) *M*_b_ (Floater)	60.10	4	*M*_bh_	60.60	6	0.50
B	*M*_o_ (Territorial) *M*_b_ (Floater)	42.39	4	*M*_bh_	44.08	6	1.69
C	*M*_o_ (Territorial) *M*_b_ (Floater)	42.39	4	*M*_h_	48.68	4	6.29
D	NA^a^	NA^a^	NA^a^	*M*_o_	38.66	1	NA^a^
E	*M*_o_ (Territorial) *M*_b_ (Floater)	29.26	4	*M*_h_	35.71	4	6.45

a Identification of territory holders is unclear, because two different male coalitions were scent marking.

The spatial tactic, mixture, and floater‐only models always correctly estimated male cheetah abundance, while the heterogeneity *M*_h_ (jackknife) and *M*_h_ (Chao) models showed less consistent results (Supporting Information Table S1). The heterogeneity *M*_h_ (jackknife) models correctly estimated abundance for two of the territories (B and E), while the heterogeneity *M*_h_ (Chao) models correctly estimated abundance for two other territories (A and C). Neither model correctly estimated abundance for territory D. Incorrect abundance estimates were always overestimates by 3.00 ± 5.61 (mean ± standard error [*SE*]) male cheetahs for the jackknife and 1.20 ± 2.17 (mean ± *SE*) male cheetahs for the Chao estimators, respectively. The spatial tactic and mixture models showed similar performance regarding precision, with each top model showing a *SE* of less than 0.001, and a range matching the abundance estimate. The estimation of *π* by the mixture models showed variation in its accuracy across territories, correctly estimating *π* for two out of four measurable territories (C and E, Supporting Information Table S1). The three models ran with program CAPTURE showed less accuracy in comparison with the two models run with MARK. Of the three models ran in program CAPTURE, the floater‐only models showed the greatest degree of precision in abundance estimates, followed by the *M*_h_ (Chao) models, while the *M*_h_ (jackknife) models showed the lowest degree of precision and performed approximately equally for the different territories (Supporting Information Table S1).

## 4 DISCUSSION

In this study, we compared the results of four closed capture–recapture heterogeneity models to true abundances of male cheetah from five territories. We demonstrated that three out of the four model types were able to accurately and precisely estimate male cheetah abundance, when camera traps were placed at predictable locations of cheetah activity. The three models were a spatial tactic model, a mixture model, and a model estimating only floater abundance. Due to the mixture model not requiring information regarding the spatial status of individuals, we recommend this model for accurately estimating the abundance of male cheetahs. The calculation of abundance at territories is the first vital step in producing population estimates across landscapes and to monitor trends in the population. The movement of floater males between multiple territories (see Section 2.1) need to be considered for the next steps when conducting population estimation. In this study, floaters visited two or three different territories, thus models calculating population estimates need to incorporate the average number of territories visited per floater and the available space for territories per region.

Spatial tactic and mixture models both gave consistently accurate and highly precise abundance estimates, with every associated *SE* being <0.001, and all ranges containing the abundance estimate itself. Such precision has not yet been recorded in closed capture–recapture studies with cheetahs. For example, two studies using the heterogeneity *M*_h_ model in CAPTURE recorded an abundance estimate of seven males with a *SE* of 1.93 and a range of 6–14 males in South Africa (Marnewick, Funston, & Karanth, 2008) or an abundance estimate of five males with a *SE* of 1.36 and a range of 5–11 males in Algeria (Belbachir, Pettorelli, Wacher, Belbachir‐bazi, & Durant, 2015). The precision of abundance estimates as in our study, coupled with the accuracy of abundance estimates, is an obvious and important advantage of spatial tactic and mixture models in MARK, over the traditionally used heterogeneity *M*_h_ model in CAPTURE.

Precision of abundance estimates in comparison with those produced by heterogeneity models used for other large felids, further highlight the favorable results of this study. Gray and Prum (2012) compared mixture models and a Huggins type gender model (comparable to the spatial tactic model used here) for leopard *Panthera pardus* and detected differences in abundance estimates between the model types. However, true abundance of leopard was unknown, thus inferences regarding the accuracy of estimates could not be made. Leopard abundance estimates had relatively large standard errors, for example, an abundance of 22.4 animals had a *SE* of 10.7 for the best fitting mixture model, and an abundance of 19.8 animals had a *SE* of 8.6 for the best fitting gender model. It was suggested that the low precision of abundance estimates were due to a low sample size of 12, combined with low detection probability. However, our study produced precise abundance estimates with lower sample sizes, and Selvan, Lyngdoh, Habib, and Gopi (2014) found mixture models to be robust even to small sample sizes when estimating tiger *Panthera tigris* abundance. A relatively high detection probability may therefore provide a better explanation for the high precision of cheetah abundance estimates, which may in turn be due to the placement of camera trap stations at marking trees.

Placement of camera trap stations at marking trees has previously been recommended as a method of increasing detection probability of cheetahs, albeit biased toward males (Boast, Reeves, & Klein, 2015; Brassine & Parker, 2015; Marker, Fabiani, & Nghikembua, 2008; Marnewick, Bothma, & Verdoorn, 2006). Camera trap success from our study was relatively high in comparison with others, ranging between 24.63 and 56.82 events/100 trap nights within a territory. In addition, it resulted in a 100% detection probability for those collared floaters entering a territory core, using a 4‐day sampling occasion. When placing camera traps at marking trees in north‐central Namibia, Marker et al. (2008) recorded 21.36 events/100 trap nights, while Marnewick et al. (2006) recorded 14.95 events/100 trap nights at a single marking tree in South Africa. Such comparisons may suggest that the use of spatial GPS data from male cheetahs to find marking trees to be key in selecting the most optimal marking trees. Other studies using combinations of roads, trails, and marking trees for camera trap placement have produced lower success rates with 0.98 events/100 trap nights in Botswana (Boast et al., 2015) and 10 events/100 trap nights in South Africa (Marnewick et al., 2008). The resulting high capture probability of male cheetahs in our study may have led to the distinct differences in capture probability between territorial and floater males. Such heterogeneity may have been masked if a different survey design was used which resulted in lower capture probabilities. Further research into the utility of such models for other species in which heterogeneity is expected, but which suffer from low capture probabilities, would be of use in gaining a better understanding of the applicability of such models across species.

We recommend mixture models as the most appropriate model for estimating male cheetah abundance, despite spatial tactic models giving the best model fit at each territory. Mixture models have the strong advantage of requiring no prior information regarding the spatial tactic of each male present in a territory, that is, it is not needed to know whether a male is a territory holder or a floater. Mixture models also produced accurate and precise abundance estimates, with no differences seen between spatial tactic top model results. In addition, mixture models were robust even when the identity of the territorial male was unclear, such as for territory D, where two different male coalitions were scent marking, maybe being in the process of sorting out territory ownership. However, the ability of mixture models to correctly estimate *π*, the probability of being a floater, was inconsistent. Thus, comparison of all individual encounter histories with each other to identify those individuals with a high frequency of detection and those with a lower frequency of detection, that is, territory holders and floater, respectively, rather than reliance on this estimate, is recommended in determining the number of floaters.

CAPTURE heterogeneity *M*_h_ models (jackknife and Chao estimators) were unable to consistently estimate true abundance, and when incorrect, overestimated abundance, although the correct abundance was contained within the estimate ranges in four of the five territories. Positive bias in abundance estimates from *M*_h_ estimators has been previously described when nearly all individuals in a survey population were captured (Chao & Huggins, 2005), as in our study. Such a situation is rare, given the typically low capture probability of target species, especially large felids, reported in published studies (Foster & Harmsen, 2012). The positive bias reported here for CAPTURE models is therefore likely due to the fact that the territorial animals, and all visiting floaters, were captured on camera traps, a result again attributed to the placement of camera trap stations at marking trees.

Due to the poor performance of the CAPTURE heterogeneity *M*_h_ model for large carnivore species, this model has recently been deemed inappropriate for the use with these species (Gray & Prum, 2012). Our study confirms this, and thus previous studies having used this model for estimating cheetah abundance, might be inaccurate and represent overestimates of abundance (e.g., Marker et al., 2008; Marnewick et al., 2008). Although true abundance was unknown in these studies and therefore inferences regarding bias cannot be made, the lack of precision in estimates clearly hampers the effective use of model results in wildlife management. In contrast to CAPTURE heterogeneity *M*_h_ models, the CAPTURE models for estimating the floater‐only abundance performed well, always estimating abundance correctly with high precision. However, like the spatial tactic models, these models require a prior knowledge of the spatial tactic of all males detected, which may not always be available.

The recently developed spatial explicit capture–recapture models (secr) were not considered appropriate for male cheetahs as these models presume the probability of detection decreases with movement away from the center of a home range (Royle et al., 2014). Such models are useful when the spatial extent of the study area needs to be defined to convert abundance into density. They produce density estimates from the onset and as a result are gaining popularity within the literature (Royle et al., 2014). However, for floater males, the probability of capture is not so much related to distance away from the center but rather from the position of territories within their home range (Figure 2, unpublished data).

The heterogeneity in capture probability for male cheetahs within a territory is largely due to floater males moving in and out of the territory, each of which is defined as a survey area, whereas territorial males spend the majority of their time within a territory (Caro, 1994). Thus, all individuals were potentially available for detection at marking trees throughout the survey period. This differs from other studies with heterogeneity in capture probability. For sex‐specific heterogeneity, for example, it was suggested that the difference between the sexes to be detected was based on the location where the camera traps were deployed, which was along roads that might have been used differently by the sexes (e.g., Gray & Prum, 2012). In such cases, the individuals of one group (sex) moved off and on the survey area and thus for certain periods were not available for detection. The distinction between the two scenarios and its ramifications for abundance modeling are unclear; however, we suggest that a scenario in which all individuals are potentially available for detection throughout the survey period are reasonably reliable.

Our study adds to the growing body of literature examining models accounting for heterogeneity in sex, social status, etc., which have been found to be a better fit than models not accounting for these differences. Both Cubaynes et al. (2010) and Cubaynes (2011), used mixture models for estimating wolf *Canis lupus* abundance using a noninvasive genetic sampling approach. These studies used two‐class mixture models, representing highly detectable (resident adults) and lowly detectable (pups, juveniles, and migrants) individuals, which may have moved out of the study area during the survey. In both studies, the heterogeneity mixture models showed better model fit than those with homogenous detection probabilities. Multievent models are another potential option for species for which capture probability or other parameters such as survival, may be influenced by the individual state. Originating from multisite models (Arnason, 1972), which were designed when individuals may be recorded successively at different sites, multievent models can be used to study repeated transitions among states, for example, breeding and nonbreeding states (Pradel, 2005). However, such models would not be considered appropriate when the studied states in a species are not reversible states, such as the spatial tactics in adult male cheetahs, which first are floaters and then, if successful, territory holders (Melzheimer et al., 2018).

Heterogeneity in detection probability is inherent to many animal populations (Lebreton et al., 1992), and examples include any species with both resident and transient or nomadic individuals, such as bottlenose dolphin *Tursiops truncatus* (Conn et al., 2011), brown hyena *Hyaena brunnea* (Mills, 1990), coyote (Larrucea et al., 2007), and many bird species including blackcaps *Sylvia atricapella* (Belda, Barba, & Monrós, 2007) and Eurasian reed warbler *Acrocephalus scirpaceus* (Clavel, Robert, Devictor, & Julliard, 2008). However, capture–recapture models assume a homogenous detection probability of individuals for population estimates (Krebs, 1999). Here, we have demonstrated the importance of modeling heterogeneity in detection probability associated with spatial tactics of male cheetahs when estimating abundance. We conclude that mixture models are most appropriate for heterogeneity in detection probability and have the advantage of requiring no prior information regarding individuals. This gives them potential application for a wide range of species for which attributes effecting detection probability, such as sex, are unknown for each individual. We recommend the application of mixture models to other species with intrasexual behavioral differences which are likely to result in heterogeneity in capture probability, particularly in situations in which model results can be compared to known abundances.

## CONFLICT OF INTEREST

None declared.

## AUTHOR CONTRIBUTIONS

SE and JM conceived the idea. MF and JM collected the data. SE and MF analyzed the data. SE wrote the manuscript. BW gave valuable input to the manuscript. All authors read and approved the final manuscript.
