# Supplementary material for: Coping with intrasexual behavioral differences: Capture–recapture abundance estimation of male cheetah
Source: Ecol Evol. 2018 Jul 30;8(18):9171–80. doi: 10.1002/ece3.4410 (PMC6194303; doi:10.1002/ece3.4410)
Supplement: Supplementary file 1 [file ECE3-8-9171-s001.docx]

**Supplementary Material**

**Figure S1:** Detection histories for each territory. Each line represents an individual, with territorial males labelled as 'T' and floater males labelled as 'F'. '1' represents a sampling occasion in which that individual was detected and '0' represents a sampling occasion in which the individual was not detected.

**Territory A**

1111101 T;

0100011 F

1001111 F

0010110 F

1000000 F

0001000 F

0001000 F

1001011 F

0100000 F

**Territory B**

1111111 T

0000100 F

1111011 F

0000010 F

1000011 F

0010000 F

0100001 F

**Territory C**

1111111 T

0011000 F

1100000 F

1111000 F

1110000 F

1100000 F

0000001 F

**Territory D (identity of territorial and floaters was unclear)**

1000001

0010000

1101000

1010001

0110000

0000100

**Territory E**

1111111 T

1010010 F

0101000 F

0010100 F

0000010 F
