# Supplementary material for: Coping with intrasexual behavioral differences: Capture–recapture abundance estimation of male cheetah
Source: Ecol Evol. 2018 Jul 30;8(18):9171–80. doi: 10.1002/ece3.4410 (PMC6194303; doi:10.1002/ece3.4410)
Supplement: Supplementary file 2 [file ECE3-8-9171-s002.docx]

**Supplementary Material**

**Table S1**: Comparison of the true abundance of territory holders and floater male units with the top model performance estimating male cheetah abundance. Spatial tactic and mixture model were run with MARK, floater only, heterogeneity Mh (jack-knife) and heterogeneity Mh (Chao) models were run with CAPTURE.

| **Territory** | **True abundance: territorial + floater (*π*)** | **Spatial tactic model: territorial (S.E., range) + floater (S.E., range)** | **Mixture model: males (S.E., range, *π***) | **Floater**  **only model: males (S.E., range)** | **M_h_ (jack-knife) model: males (S.E., range)** | **M_h_ (Chao) model: males (S.E., range)** |
| --- | --- | --- | --- | --- | --- | --- |
| A | 1+8 (0.89) | 1 (<0.001, 1-1) + 8 (<0.001, 8-8) | 9 (<0.001, 9-9, 0.61) | 8 (0.77, 8-8) | 22 (7.33, 14-45) | 9 (0.00, 9-9) |
| B | 1+6 (0.86) | 1 (<0.001, 1-1) + 6 (<0.001, 6-6) | 7 (<0.001, 7-7, 0.71) | 6 (0.69, 6-6) | 7 (2.83, 7-20) | 12 (7.19, 8-47) |
| C | 1+6 (0.86) | 1 (0.00, 1-1) + 6 (0.00, 6-6) | 7 (0.00, 7-7, 0.86) | 6 (0.69, 6-6) | 8 (1.33, 8-14) | 7 (0.54, 7-10) |
| D | Total: 6 males | NA | 6 (<0.001, 6-6, NA) | NA | 8 (1.85, 7-15) | 7 (1.87, 7-17) |
| E | 1+4 (0.80) | 1 (<0.001, 1-1) + 4 (0.00, 4-4) | 5 (0.00, 5-5, 0.80) | 4 (0.77, 4-4) | 5 (0.73, 5-9) | 6 (1.31, 5-9) |

*π: probability of any individual within the population being a floater male*
